# Italian observational study on HPV infection, E6, and p16 expression in men with penile cancer

**DOI:** 10.1186/s12985-020-01424-9

**Published:** 2020-10-22

**Authors:** Narcisa Muresu, Giovanni Sotgiu, Laura Saderi, Illari Sechi, Antonio Cossu, Vincenzo Marras, Marta Meloni, Marianna Martinelli, Clementina Cocuzza, Francesco Tanda, Andrea Piana

**Affiliations:** 1 Department of Medical, Surgical and Experimental Sciences, University of Sassari, Via Padre Manzella, 4, 07100 Sassari, Italy; 2 Department of Biomedical Sciences, University of Sassari, 07100 Sassari, Italy; 3 Department of Medicine and Surgery, University of Milano-Bicocca, 20900 Monza, Italy

**Keywords:** HPV, Penile cancer, E6, p16, HPV-DNA

## Abstract

**Background:**

Human Papillomavirus (HPV) infection is one of the most important causes of cancer. It can play a role in cervical and extra-cervical cancers. Penile cancer is rare, even if an increasing trend was recently reported. Aim of the present study was to assess the prevalence and distribution of HPV genotypes in cases of penile cancer diagnosed in Sardinia, Italy. Surrogate markers of HPV infection (*i.e.*, E6 and p16 genes) were also evaluated in all cases.

**Methods:**

An observational, retrospective study which recruited all cases of penile cancer diagnosed between 2002 and 2019 at a tertiary care hospital in Sardinia, Italy, was carried out. HPV-DNA detection and genotyping were performed by Real-time PCR. Specimens were tested for oncogene E6 mRNA and for p16(INK4a) expression.

**Results:**

HPV prevalence was 28.1% (9/32); HPV-16 was the most prevalent genotype (7/9, 77.8%). p16INK4a positivity was found in 66.7% of the samples with a statistically significant difference between HPV-positive and -negative groups. E6-transcript was detected in 71% of the HPV-16 positive samples. The overall survival was not statistically different between HPV-positives and -negatives.

**Discussion:**

The present study confirms the etiologic role of HPV in penile cancer and supports the adoption of vaccination strategies in men and women. Further studies should clarify the diagnostic and prognostic role of E6 and p16 proteins.

**Conclusion:**

HPV infection can favor the occurrence of penile cancer, whose diagnosis and prognosis could be improved with the implementation of validated molecular techniques.

## Introduction

Human Papillomavirus (HPV) is one of the leading causes of cancer, with ~ 690,000 new annual cases worldwide. HPV vaccination and PAP-testing (*i.e.,* Papanicolau test) have significantly reduced the incidence of cervical carcinoma; however, an increasing trend of other HPV-related cancers (e.g., anogenital and head and neck cancers) has been described worldwide. It has been estimated that > 90%, > 50%, 77%, and 25% of anal, penile, vaginal, and vulvar cancers are attributable to HPV, respectively [1].

Penile cancer is a rare disease in the general population, accounting for < 0.5% of all diagnosed cancers [2]. Several risk factors of penile cancer have been found, including phimosis, poor hygiene, smoking, chronic genital inflammation, and sexually transmitted infections [3]. Several studies reported on HPV infection in more than 20% of penile cancer cases, suggesting a role of HPV in penile carcinogenesis [4]. However, HPV detection in patients with penile carcinoma significantly ranges from ~ 1 to ~ 73: this wide variability could depend on population target, tumor stage, and HPV detection methods [4].

Malignancy is associated with an over-expression of E6/E7 and p16 genes and, consequently, with a deregulation of the cell cycle through the inhibition of oncosuppressors p53 and pRb. However, those translational findings were proved in studies on cervical cancer. The identification of the mechanisms behind the occurrence of penile cancer could provide key targets for prevention and therapy.

Aim of the present study was to assess HPV prevalence and genotype distribution in the Sardinian region, Italy. Furthermore, the diagnostic and prognostic role of E6 and p16 proteins was evaluated in HPV infected men.

## Methods

An observational, retrospective, single-center study was carried out at a tertiary-care university hospital in Sassari, Italy. Patients diagnosed with penile cancer were retrospectively recruited from 2002 to 2019.

Samples of penile cancer were collected from the archived formalin-fixed paraffin-embedded (FFPE) specimens: they underwent histopathology and p16 immunohistochemistry. In total, 10 consecutive sections of 3 µm were cut from FFPE blocks. Hematoxylin and eosin stained sections were analyzed by two pathologists to confirm diagnosis and tumor staging, following the recommendations of the classification system of the American Joint Committee on Cancer (AJCCC) [5]. Immunohistochemistry was performed for p16 on formalin-fixed paraffin-embedded tissue sections using the kit CINtec p16 Histology (Ventana Medical Systems, Inc. Tucson, AZ, USA), following the manufacturer’s protocol [6].

Molecular analyses were carried out as reported in another publication [7]. After deparaffinization, acid nucleic extraction was performed using a commercially available extraction kit (AllPrep DNA/RNAFFPE Kit, Qiagen, Hilden, Germany) in accordance with the manufacturer’s instructions [8]. HPV-DNA detection and genotyping was conducted using a multiplex polymerase chain reaction (PCR) using the commercial kit AnyplexTM II HPV28 Detection (Seegene, Seoul, South Korea) [9], which included 19 high (i.e.,16, 18, 26, 31, 33, 35, 39, 45, 51, 52, 53, 56, 58, 59, 66, 68, 69, 73, and 82) and 9 low-risk (i.e., 6, 11, 40, 42,43, 44, 54, 61, 70) HPV genotypes.

HPV-16 positive samples were tested for E6-mRNA by reverse transcriptase polymerase chain reaction (RT-PCR), using a set of primers designed by Sotlar et al. [10] for the U1-gene (forward5′-cagagctgcaaacaactatacatgatata-3′, reverse 5′-gttaatacacctcacgtcgcagta-3′) and for the E6 transcript(forward 5′-gaagatcaagaaggatgagctaaaaa-3′, reverse 5′-tgggagaagatggcgtacag-3′).A cDNA synthesis was carried out using a Quantitect Reverse Transcription Kit(QIAGEN co. 205312) following the manufacturer’s recommendations [11]. Qualitative real-time PCRwas carried out using SYBR Green JumpStart TaqReadyMix (Cat. Num. S9194. SIGMA-ALDRICH,St Louis, MO, USA) [12]. Nuclease-free water and previously tested HPV16-positive liquid-based cytology samples were added in each experiment as negative and positive controls, respectively. Ethical approval was waived based on the observational study design (not requested by the Italian law according to the GU No. 76 31/Mar/2008).

An ad hoc electronic form was prepared to collect demographic, clinical, and epidemiological variables. The following variables were retrieved from medical files: age, year of diagnosis, comorbidities, histopathological classification, HPV-DNA positivity, HPV genotype, p16INK4a and E6 proteins expression. Qualitative variables were summarized with absolute and relative (percentages) frequencies, whereas quantitative variables were summarized with means (standard deviations, SD). Student t test was computed to detect statistical differences for quantitative variables following abnormal distribution. Chi-squared and Fisher exact test were computed to assess differences for qualitative variables. Kaplan–Meier survival analysis was performed to assess the mortality in different subgroups. A two-tailed p-value less than 0.05 was considered statistically significant. All statistical analyses were performed with the statistical software STATA version 16 (StatsCorp, Texas, USA).

## Results

Thirty-two cases of penile cancer were diagnosed between 2002 and 2019. The mean (SD) age was 68 (11.6) years (Table 1). All recruited cases were squamous cell carcinoma (SCC), mainly localized in the glans (24/32, 75.0%); ~ 60% (19/32) and 25% (8/32) were well-and moderate differentiated, respectively.

**Table 1:** Epidemiological, clinical, and virologic characteristics of the study sample

Mean age (SD), years	68.0 (11.6)
Age ≥ 55 years, n (%)	28/32 (87.5)
*Civil status, n (%)*	
Married	13/17 (76.5)
Single	2/17 (11.8)
Widower	2/17 (11.8)
*Occupational status, n (%)*	
Employed	7 (38.9)
Retired	11 (61.1)
Familiarity for carcinoma, n (%)	1/15 (6.7)
Suspected diagnosis of carcinoma, n (%)	23/24 (92.0)
Cancer diagnosed before 2010, n (%)	10.32 (31.3)
*Anatomic site, n (%)*	
Glans	24/32 (75.0)
Penis	8/32 (25.0)
*Histopathological results, n (%)*	
Squamous Cell Carcinoma	30/32 (93.8)
Hyperplasia	1/32 (3.1)
Fibrosis	1/32 (3.1)
Well differentiated, n (%)	19/32 (59.4)
Moderately differentiated, n (%)	8/32 (25.0)
Poorly differentiated, n (%)	5/32 (15.6)
*Histopathological grading (AJCC), n (%)*	
G1	19/32 (59.4)
G2	8/32 (25.0)
G3	5/32 (15.6)
*Primary tumor (T) (AJCC), n (%)*	
Tis	3/32 (9.4)
T1	18/32 (56.3)
T1a	2/32 (6.3)
T1b	1/32 (3.1)
T2	1/32 (3.1)
T3	4/32 (12.5)
NC	3/32 (9.4)
HPV positivity, n (%)	9/32 (28.1)
HPV-16	7/32 (21.9)
HPV-56	2/32 (6.3)
HPV-35	1/32 (3.1)
p-16 immunohistochemistry positivity, n (%)	7/32 (21.9)
E6 gene positivity, n (%)	5/7 (71.4)
Outcome, died	14/32 (43.8)

^*^Denominators of the collected variables can change based on the available information retrieved from the medical files

HPV infection was diagnosed in 28.1% (9/32) samples. HPV-16 was the most frequent genotype (7/9, 77.8%), followed by HPV-56 (1/32, 3.1%), and HPV-35 (1/32, 3.1%). Only one patient had multiple infections, caused by the genotypes HPV-16 and -35. No statistically significant differences were found for HPV infection and genotype distribution according to age and year of diagnosis.

**Fig. 1:**
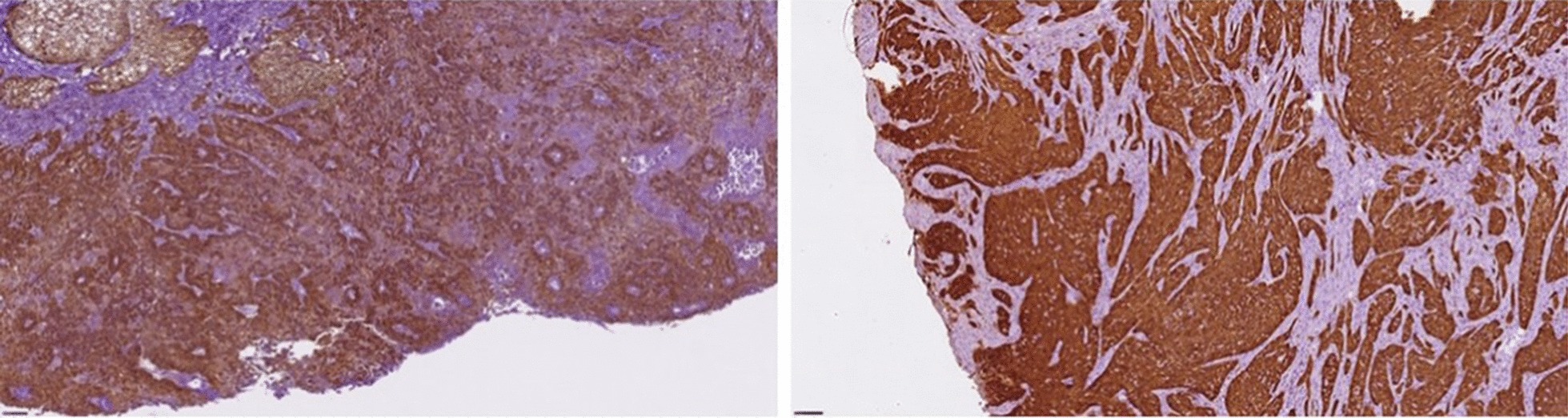
Immunohistochemical staining for p16 in HPV-positive and -negative sample

Immunohistochemical staining for p16 protein was positive in ~ 22% of specimens (7/32); 85.7% (6/7) of them were HPV-DNA positive (*p *value: 0.01) (Fig. 1).

E6 transcript was found in 71.4% (5/7) of HPV-16 positive samples (Table 2).

**Table 2:** Comparison of demographic, epidemiological and clinical variables between positive and negative HPV patients

	HPV negativity	HPV positivity	*P* value
Mean age (SD), years	69.6 (10.6)	64.1 (13.7)	0.24
Age ≥ 55 years, n (%)	21/23 (91.3)	7/9 (77.8)	0.56
*Civil status, n (%)*			
Married	8/12 (66.7)	5/5 (100.0)	0.34
Single	2/12 (16.7)	0/5 (0.0)	
Widower	2/12 (16.7)	0/5 (0.0)	
*Occupational status, n (%)*			
Employed	5/12 (41.7)	2/6 (33.3)	0.73
Retired	7/12 (58.3)	4/6 (66.7)	
Familiarity for carcinoma, n (%)	1/11 (9.1)	0/4 (0.0)	1.0
Suspected diagnosis of carcinoma, n (%)	16/17 (94.1)	7/8 (87.5)	1.0
Cancer diagnosed before 2010, n (%)	7/23 (30.4)	3/9 (33.3)	1.0
*Anatomic site, n (%)*			
Glans	18/23 (78.3)	6/9 (66.7)	1.0
Penis	5/23 (21.7)	3/9 (33.3)	1.0
*Histopathological results, n (%)*			
Squamous Cell Carcinoma	23/23 (100.0)	7/9 (77.8)	0.07
Hyperplasia	0/23 (0.0)	1/9 (11.1)	
Fibrosis	0/23 (0.0)	0/9 (0.0)	
Well differentiated, n (%)	15/23 (65.2)	4/9 (44.4)	0.43
Moderately differentiated, n (%)	6/23 (26.1)	2/9 (22.2)	1.0
Poorly differentiated, n (%)	2/23 (8.7)	3/9 (33.3)	0.12
*Histopathological grading (AJCC), n (%)*			
G1	15/23 (65.2)	4/9 (44.4)	0.25
G2	6/23 (26.1)	2/9 (22.2)	
G3	2/23 (8.7)	3/9 (33.3)	
*Primary tumour (T) (AJCC), n (%)*			
Tis	2/23 (8.7)	1/9 (11.1)	0.83
T1	13/23 (56.5)	5/9 (55.6)	
T1a	2/23 (8.7)	0/9 (0.0)	
T1b	1/23 (4.4)	0/9 (0.0)	
T2	1/23 (4.4)	0/9 (0.0)	
T3	3/23 (13.0)	1/9 (11.1)	
NC	1/23 (4.4)	2/9 (22.2)	
p-16 immunohistochemistry positivity, n (%)	1/23 (4.4)	6/9 (66.7)	0.001
E6 gene positivity, n (%)	0/0 (0.0)	5/7 (71.4)	–
Outcome, died	11/23 (47.8)	3/9 (33.3)	0.69

**Fig. 2:**
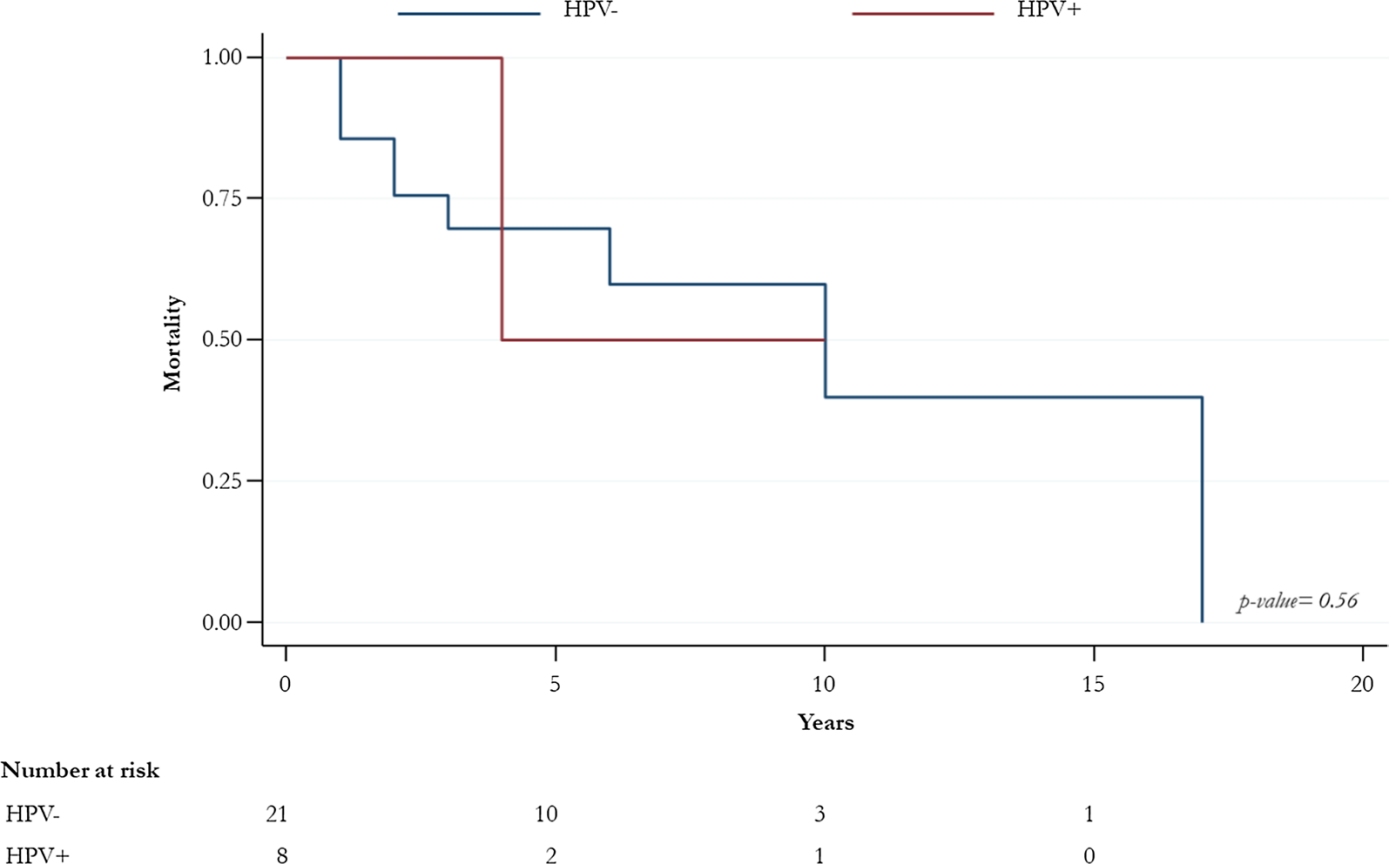
Overall survival for HPV-positive and -negative patients

43.8% (14/32) of patients died, with only 21.4% (3/14) HPV-infected. However, no statistically significant differences were observed for the outcome mortality between HPV-positives and -negatives (Fig. 2).

A higher risk of death was found for poorly o moderately differentiated (G2, G3) cases (OR: 6.3, 95% CI: 1.3–30.0; *p* value: 0.02) (Table 3).

**Table 3:** Logistic regression analysis to assess the relationship between demographic, epidemiological and clinical variables and mortality

	OR (95% CI)	*p* value
Age, years	1.0 (0.9–1.1)	0.99
Age ≥ 55 years	0.2 (0.0–2.4)	0.21
Married	0.6 (0.1–6.0)	0.68
Employed	1.6 (0.2–10.8)	0.63
Anatomic site, Penis	0.3 (0.1–1-2.0)	0.23
Squamous Cell Carcinoma	0.8 (0.0–13.4)	0.85
Well differentiated, n (%)	0.2 (0.0–0.8)	0.02
Moderately differentiated, n (%)	2.8 (0.5–14.5)	0.23
Poorly differentiated, n (%)	6.8 (0.7–69.6)	0.11
Histopathological grading 2–3	6.3 (1.3–30.0)	0.02
HPV positivity, n (%)	0.6 (0.1–2.7)	0.46
HPV-16	1.0 (0.2–5.2)	0.96
HPV-56	1.3 (0.1–22.9)	0.85
HPV-35	–	–
p-16 immunohistochemistry positivity, n (%)	1.0 (0.2–5.2)	0.96

A total of fourteen patients showed comorbidities (*i.e.*, 18 medical conditions). The most prevalent comorbidities were inguinal lymph node metastasis (4/18, 22.2%), colic adenoma (3/18, 16.7%), pulmonary metastasis (2/18, 11.1%), and squamous cell carcinoma (2/18, 11.1%). Three samples (*i.e.*, two inguinal lymph node metastasis and one pulmonary metastasis) were HPV-16 and E6 transcript positive in two patients (2/14, 14.3%) who showed HPV-positive penile samples.

## Discussion

To the best of our knowledge, this is the first study on HPV infection in patients with penile cancer carried out in Sardinia, Italy. Furthermore, the present study evaluated the diagnostic and prognostic role of E6 and p16 proteins in patients with HPV-related cancer.

The HPV prevalence in patients with penile cancer was 28.1%, with HPV-16 being the most prevalent genotype, as previously described in cervical and extra-cervical HPV-associated cancers [7, 13].

A recent systematic review carried out by Olesen et al. recruiting 4000 patients with penile cancer [14] reported on a HPV infection prevalence ranging from 11.6 to 100% (mean prevalence: ~ 50%). In contrast, Alemany et al*.* found an HPV-positivity of 33% in cases of penile cancers and 87% in high-grade squamous intraepithelial lesions (HGSILs) [15]. These epidemiological results show the wide variability of prevalence estimates reported in HPV-related cancers; it is unclear if this epidemiological heterogeneity depends on recruited populations, histological classification, or HPV detection methods.

The p16 immunohistochemistryshowed a positivity of 22% with a statistically significant difference between HPV-positive and -negative cases: this finding could suggest its potential adoption for the diagnosis of HPV infection in patients with penile cancer [16]. Only one p16-positive case was HPV-DNA negative, which could be explained by the infection caused by one genotype not included in the molecular panel we used. Although the agreement among HPV-DNA, p16, and E6 was good, we would suggest the adoption of at least two methods to increase the diagnostic accuracy.

No statistically significant differences were found between HPV-positive and -negative groups when we compared epidemiological and clinical variables. The poor sample size could not potentially help assess differences which occur in the population. However, our data confirmed that the SCC is the most frequent histological subtype, mainly in persons aged ≥ 55 years [17].

It is scientifically unclear a better prognosis has been found in HPV-positive cases [18]. However, we did not find any statistically significant differences in terms of survival between HPV-positives and -negatives.

It is confirmed a high risk of secondary lesions, mainly inguinal and pelvic lymphnode metastasis, whereas only in 1–10% of the cases other anatomic sites are affected [19]. A striking finding was the HPV-16 positivity in metastatic specimens of two patients, who developed metastasis in inguinal lymphnodes and in the lungs. All specimens were positive for the same genotype detected in the primary tumor. Our results could suggest a role of HPV in the occurrence of secondary malignancies through the lymphatic circulation, as reported by several authors [20, 21], as well as a higher risk of secondary malignancies in patients with HPV-positive lymph node metastasis, regardless their gender [20].

Although our study has described for the first time the etiologic role of HPV in penile cancer in an Italian region, some limitations should be highlighted. First, the small sample size could be associated with the absence of statistically significant differences and associations between the collected epidemiological variables and HPV infection. Moreover, missing demographic and clinical data linked to the retrospective nature of the study did not provide a comprehensive picture. The lower prevalence of E6 transcript-positive cases, if compared with HPV-DNA positivity, could be partially explain by the RNA degradation often reported in FFPE samples; fresh samples can be used in future studies to assess the diagnostic and prognostic role of those molecular markers in HPV-related medical conditions. Furthermore, we could not assess the effectiveness of therapies prescribed the attending physicians.

## Conclusions

Our study, the first of its kind in the Sardinian region, confirms the role played by HPV in the etiology of penile cancer. Moreover, the diagnostic and prognostic role of E6 and p16 proteins should be further investigated in larger studies. The implementation and scale-up of primary preventive strategies (*i.e.,* HPV vaccination) in men and women could rapidly decrease the national burden of HPV-related diseases. Moreover, further studies are needed to assess the role of HPV infection in secondary malignancies and the potential therapeutic role of HPV vaccines in the reduction of the risk of recurrence. Larger multicenter studies and standardization of molecular methods could be useful to validate the diagnostic and prognostic process of biomarkers in cases of penile cancers.

## Data Availability

Dataset is available in case it is requested for motivated reasons.

## Abbreviations

AJCCC: American Joint Committee on Cancer
FFPE: Formalin-fixed paraffin embedded
HPV: Human Papilloma Virus
SCC: Squamous cell carcinoma
SD: Standard deviation
